# The Heat Shock Protein 90 Inhibitor, AT13387, Protects the Alveolo-Capillary Barrier and Prevents HCl-Induced Chronic Lung Injury and Pulmonary Fibrosis

**DOI:** 10.3390/cells11061046

**Published:** 2022-03-19

**Authors:** Ruben M. L. Colunga Biancatelli, Pavel Solopov, Christiana Dimitropoulou, Betsy Gregory, Tierney Day, John D. Catravas

**Affiliations:** 1 Frank Reidy Research Center for Bioelectrics, Old Dominion University, Norfolk, VA 23508, USA; psolopov@odu.edu (P.S.); cdimitro@odu.edu (C.D.); bgregory@odu.edu (B.G.); tday@odu.edu (T.D.); jcatrava@odu.edu (J.D.C.); 2 School of Medical Diagnostics & Translational Sciences, College of Health Sciences, Old Dominion University, Norfolk, VA 23508, USA

**Keywords:** HCl exposure, endothelial dysfunction, pulmonary fibrosis, heat shock proteins, HSP90 inhibitors, AT13387

## Abstract

Hydrochloric acid (HCl) exposure causes asthma-like conditions, reactive airways dysfunction syndrome, and pulmonary fibrosis. Heat Shock Protein 90 (HSP90) is a molecular chaperone that regulates multiple cellular processes. HSP90 inhibitors are undergoing clinical trials for cancer and are also being studied in various pre-clinical settings for their anti-inflammatory and anti-fibrotic effects. Here we investigated the ability of the heat shock protein 90 (HSP90) inhibitor AT13387 to prevent chronic lung injury induced by exposure to HCl in vivo and its protective role in the endothelial barrier in vitro. We instilled C57Bl/6J mice with 0.1N HCl (2 µL/g body weight, intratracheally) and after 24 h began treatment with vehicle or AT13387 (10 or 15 mg/kg, SC), administered 3×/week; we analyzed histological, functional, and molecular markers 30 days after HCl. In addition, we monitored transendothelial electrical resistance (TER) and protein expression in a monolayer of human lung microvascular endothelial cells (HLMVEC) exposed to HCl (0.02 N) and treated with vehicle or AT13387 (2 µM). HCl provoked persistent alveolar inflammation; activation of profibrotic pathways (MAPK/ERK, HSP90); increased deposition of collagen, fibronectin and elastin; histological evidence of fibrosis; and a decline in lung function reflected in a downward shift in pressure–volume curves, increased respiratory system resistance (Rrs), elastance (Ers), tissue damping (G), and hyperresponsiveness to methacholine. Treatment with 15 mg/kg AT13387reduced alveolar inflammation, fibrosis, and NLRP3 staining; blocked activation of ERK and HSP90; and attenuated the deposition of collagen and the development of chronic lung injury and airway hyperreactivity. In vitro, AT13387 prevented HCl-induced loss of barrier function and AKT, ERK, and ROCK1 activation, and restored HSP70 and cofilin expression. The HSP90 inhibitor, AT13387, represents a promising drug candidate for chronic lung injury that can be administered subcutaneously in the field, and at low, non-toxic doses.

## 1. Introduction

Heat Shock Proteins (HSPs) are molecular chaperones that assist the folding, stabilization and activity, but when necessary also participate prominently in the degradation, of a high number of “client proteins” [1]. The HSP family consists of multiple proteins with specific and diverse functions, classified by molecular weight. Heat Shock Protein 90 (HSP90) is the most abundant of these proteins, comprising 1–2% of the overall cellular proteins under basal conditions, and able to reach 5–10% during stress. HSP90 stabilizes folded proteins, thus promoting the proper signaling of crucial pathways involved in cell survival from environmental stressors such as hypoxia, high temperature, and oxidative stress [2]. Recent reports suggest that by disrupting protein stability and blocking specific cellular responses, HSP90 inhibition may represent a novel therapeutic approach to pulmonary fibrosis [3,4].

HCl is a chemical produced in massive quantities (20 million metric tons/year [5]) and employed in oil, gas, and steel industries, in scientific laboratories, and in swimming pool maintenance. Due to its wide use, spills occur frequently and cause accidental exposures. In addition, HCl and its more volatile derivate, chlorine (Cl), have been utilized in chemical warfare since World War II, and recently in the Syrian province of Aleppo, causing multiple casualties [6]. Acutely, HCl evokes strong inflammation in the eyes and airways, with shortness of breath, difficulty in breathing and, depending on dose and duration of exposure, acute respiratory distress syndrome (ARDS) and death [7,8]. Furthermore, after a single exposure, a persistent mild inflammatory and pro-fibrotic response is observed, leading to chronic injuries, such as reactive airways dysfunction syndrome (RADS), asthma-like conditions, and pulmonary fibrosis [8,9,10].

There are no FDA-recommended drugs to prevent dangerous complications from HCl exposure. We have previously reported that the HSP90 inhibitor AUY-922 prevented both HCl and nitrogen mustard-induced long-term pulmonary injury and fibrosis [11,12]. These beneficial effects have been observed by us and others, as HSP90 is a critical stabilizer of transforming growth factor-β (TGF-β), the leading cytokine in pulmonary fibrosis [13,14]. AUY-922, however, requires intraperitoneal or intravenous administration and thus is difficult to administer outside hospital settings. Therefore, we investigated the antidotal properties of a newer HSP90 inhibitor, AT13387 (also known as Onalespib), which has a longer half-life [15] and can be administered subcutaneously in out-of-hospital, emergency situations.

## 2. Materials and Methods

### 2.1. Materials

HCl, ACS grade, methacholine USP grade, RIPA buffer, and protease inhibitor cocktail were obtained from Sigma-Aldrich Corporation (St. Louis, MO, USA). Socumb (pentobarbital) USP grade, Anased (xylazine) USP grade, and Ketaset (ketamine) USP grade were supplied by Henry Schein Animal Health (Pittsburg, PA, USA). Formaldehyde (10%) was purchased from Thermo Fisher Scientific (Waltham, MA, USA), the BCA Protein assay kit from Pierce Co. (Rockford, IL, USA), EDTA and Western blot membranes from GE Healthcare (Chicago, IL, USA), TRIzol and SuperScript VILO reverse transcriptase kit from Invitrogen (Carlsbad, CA, USA), RNeasy Mini Kit from Qiagen (Hilden, Germany), and SYBR Green Master Mix from Applied Biosystems (Carlsbad, CA, USA). All primers used for real-time quantitative PCR were purchased from Integrated DNA Technologies, Inc. (Coralville, IA, USA). SDS-PAGE, ProtoGel (30% acrylamide mix), and TEMED were from National Diagnostics (Atlanta, GA, USA), Tris-HCl buffer from Teknova (Hollister, CA, USA), 10% SDS and ammonium persulfate from Thermo Fisher Scientific, and Protein Dual Color Standards and Tricine Sample Buffer from Bio-Rad Laboratories (Hercules, CA, USA). All antibodies were purchased from reputable commercial sources and have published immunospecificity data. For antibodies used in Western blots, rabbit total and phosphorylated (1:1000; ERK1/2, HSP90, HSP70, AKT, Cofilin, ROCK1), antibodies were obtained from Cell Signaling Technology, Inc. (Danvers, MA, USA), mouse monoclonal anti-beta actin from Sigma-Aldrich Corporation (1:1000), and IRDye 800CW Goat anti-rabbit and IRDye 680RD Goat anti-mouse antibodies (1:5000) from LI-COR Biosciences (Lincoln, NE, USA).

### 2.2. Ethical Statement

All animal studies were approved by Old Dominion University IACUC, adhere to the principles of animal experimentation as published by the American Physiological Society, and were carried out in Biosafety Level 2 (BSL-2) and Animal Biosafety Level 2 (ABSL-2) facilities at the Frank Reidy Research Center for Bioelectrics in Norfolk, Virginia.

### 2.3. Animals

Adult male C57Bl/6J mice (Jackson Laboratories, Bar Harbor, ME, USA; 8–10 weeks old, 24–28 g body weight) were housed under pathogen-free conditions. Mice were intratracheally instilled with vehicle or HCl (0.1 N) and treated after 24 h with either vehicle (10% DMSO in corn oil) or with the HSP90 inhibitor AT13387 (10 or 15 mg/kg). Mice were then divided into four treatment groups: (1) mice that were exposed to the vehicle (saline), (2) mice that were exposed to 0.1 N HCl and treated with the vehicle (10% DMSO in corn oil) 3 times/week, (3) mice that were exposed to 0.1 N HCl and treated with AT13387 15 mg/kg, 3 times/week, and (4) mice that were exposed to 0.1 N HCl and treated with AT13387, 10 mg/kg, 3 times/week. On day 0, the mice were anesthetized with intraperitoneal (i.p.) injections of xylazine (6 mg/kg) and ketamine (60 mg/kg). An i.p. bolus of sterile normal saline (10 µL/g) was given as pre-emptive fluid resuscitation. After cleaning and disinfecting the surgical field, a small neck skin incision (~1 cm) was made and the salivary glands were separated to visualize the trachea. Mice were suspended vertically from their incisors and a fine, (20–25 G) plastic catheter was advanced into the trachea (~1.5 cm) in such a way that it could be seen through the walls of the trachea. Freshly prepared HCl solution (groups 2–4) or sterile saline (group 1) was instilled (2 µL/g body weight) and flushed with 100 µL air. The catheter was withdrawn, the neck incision closed by surgical adhesive Vetbond, and the animals were placed in the ventral position in a small chamber on top of a heating pad under supplemental oxygen (slowly weaned from 100 to 21% O_2_) and observed for the next 4 h for signs of respiratory distress. Mice were returned to their home-cages (five mice/cage) and monitored daily for abnormal physical appearances. On day 30, they were either anesthetized for lung function measurements or euthanized, and bronchoalveolar lavage fluid (BALF) and lungs were collected for protein and mRNA analyses or fixed for histological analysis.

### 2.4. Bronchoalveolar Lavage Fluid (BALF) White Blood Cell Number and Total Protein Concentration

BALF was obtained by instilling and withdrawing sterile 1x PBS (1 mL) via a tracheal cannula. The fluid was centrifuged at 2500× *g* for 10 min and the supernatant was aspirated and stored at −80 °C. The cell pellet was re-suspended in 1 mL sterile PBS. The total number of white blood cells (WBC) was determined using a hemocytometer. For differential cell counts, within one hour after collecting BALF, the samples were mixed gently and the cell suspensions were spun onto glass slides using a Cytospin 4 centrifuge (Thermo Fisher, Waltham, MA, USA) set at 300 rpm for 10 min. The slides were stained using the May–Grunwald–Giemsa staining protocol (Differential Quick Staining Kit, Electron Microscopy Sciences, Hatfield, PA, USA), and a coverslip was mounted. A minimum of 400 cells were identified and counted under light microscopy (Olympus BX-46, Tokyo, Japan). After the fluid was centrifuged at 2500× *g* for 10 min, the supernatant was collected for estimation of total protein. The total protein concentration was determined using a micro bicinchoninic acid (BCA) Protein Assay Kit according to the manufacturer’s instructions.

### 2.5. Histopathology, Immunohistochemistry and Lung Fibrosis Scoring

The mice were euthanized and their lungs were fixed by intratracheal instillation. A small transverse incision was made in the middle of the trachea, and the lungs were instilled and inflated with 10% formaldehyde solution to a pressure of 15 cm H_2_O through a 20 G catheter. The trachea was then ligated with sutures and the lungs were removed from the thorax and placed in 10% formaldehyde solution for 72 h. Mid-transverse slices were made from the formalin-fixed lung tissue samples and were embedded in paraffin. Sections 5 µm thick were prepared from the blocks and stained with Masson’s trichrome stain and for NLRP3. The NLPR3 antibody was used at a dilution of 1:1280, and staining was performed using a standard protocol with HRP conjugated secondary antibody. Ten slides were stained, and ten slides were used as negative controls where the primary antibody was omitted. Positive control tissue (mouse esophagus) stained positive. Ten randomly selected fields from each slide were examined under 10× and 20× magnification. All the slides were scored according to the Ashcroft score method in order to estimate the severity of pulmonary fibrosis [16]. The observer was blinded to the treatment.

### 2.6. Tissue Collection

Immediately after euthanasia, the chest was opened, blood was collected from the heart through the right ventricle, and the pulmonary circulation was flushed out with sterile PBS containing EDTA. The lungs were dissected from the thorax, snap-frozen in liquid nitrogen, and kept at −80 °C for later analysis.

### 2.7. Western Blot Analysis

Proteins in lung tissue homogenates were extracted from frozen lungs by sonication (50% amplitude, 3 times for 10 s) in ice-cold RIPA buffer with added protease inhibitor cocktail (100:1). The protein lysates were gently mixed under agitation for 3 h at 4 °C, and then centrifuged twice at 14,000× *g* for 10 min. The supernatants were combined, and total protein concentration was determined using the micro-BCA assay. Equal amounts of protein from all lysates were used for Western blot analysis. The samples were first mixed with Tricine Sample Buffer 1:1, then boiled for 5 min, then separated on a 10–12% polyacrylamide SDS gel by electrophoresis. Separated proteins were then transferred to a nitrocellulose membrane and incubated with the appropriate primary antibody, followed by incubation with the secondary antibody and detection by digital fluorescence imaging (LI-COR Odyssey CLx, Dallas, TX, USA). Beta-actin was used as the loading control. ImageJ software v.1.8.0 was used to perform densitometric quantification of the bands (http://imagej.nih.gov/ij (accessed on 20 June 2021); National Institutes of Health, Bethesda, MD, USA). For ERK and p-ERK, both bands were quantified together. Membranes were stripped in a stripping buffer for 20 min, blocked, and incubated with other primary and secondary antibodies.

### 2.8. RNA Isolation and Quantitative Real-Time PCR (qPCR)

Lung tissue, stored in an RNAlater solution, was dried and homogenized in TRIzol^®^ (Invitrogen, Carlsbad, CA, USA) followed by a cleaning step using the RNeasy Mini Kit. The purified RNA was transcribed into cDNA using the SuperScriptTM IV VILO Reverse transcriptase kit (Invitrogen, Carlsbad, CA, USA) and was analyzed by real-time qPCR with SYBR Green Master Mix on a StepOne Plus Real-Time PCR System (Applied Biosystems v.2.3). The results were evaluated using the standard curve method and were expressed as the fold of the control values, normalized to β-actin. Specifically designed primer pairs and qPCR conditions were applied to selectively determine the expression of mouse β-actin, fibronectin, collagen 1α2, and elastin, as previously described [17,18]. Fibronectin, elastin, collagen 1α2, and β-actin primers were as follows: Fibronectin F: GAA GTC GCA AGG AAA CAA GC; Fibronectin R: GTT GTA GGT GAA CGG GAG GA; elastin F: GGA GTT CCC GGT GGA GTC TAT T; elastin R: ACC AGG AAT GCC ACC AAC ACC TG; Collagen 1α2 F: GAA GCA CGT CTG GTT TGG A; Collagen 1α2 R: ACT CGA ACG GGA ATC CAT C; Beta actin F: 50-CCC CTG AGG AGC ACC GTG TG-30; and Beta-actin R: 50-ATG GCT GGG GTG TTG AAG GT-30.

### 2.9. Lung Mechanics Measurements

The mice were anesthetized with pentobarbital (90 mg/kg, i.p.), tracheostomized with a metal 1.2 mm (internal diameter) cannula, and connected to a FlexiVent small animal ventilator (SCIREQ Inc., Montreal, QC, Canada), as previously published [19]. Ventilation was performed at a tidal volume of 10 mL/kg and a respiratory rate of 150/min. A 15-min stabilization period was allowed before any measurements began. Firstly, following a deep inflation, resting static compliance (Cst, mean of three values) and pressure volume relationships (PV curves) were estimated by stepwise increasing airway pressure to 30 cm H_2_O and then reversing the process. Both parameters reflect the intrinsic elasticity of the lungs and are either reduced (Cst) or shifted to the right (PV curves) in fibrosis. Secondly, Snapshot-150 and Quick Prime-3 manoeuvres were performed. Respiratory system resistance (Rrs) and elastance (Ers), reflecting the behaviour of the entire respiratory system (peripheral and conducting airways, chest wall, and parenchyma); Newtonian resistance (Rn); tissue damping (G); inspiratory capacity (A); and the curvature of the PV loops (K) reflecting resistance of the large, conducting airways, parenchymal stiffness, and changes in inspiratory gas dynamics, were calculated, and are presented as a mean of 12 recordings.

### 2.10. Cell Culture and Protein Extraction

In-house harvested human lung microvascular endothelial cells (HLMVEC) were maintained in M199 media supplemented with 20% FBS and antibiotics/antimycotics, as described previously [20]. HLMVEC were cultured in 100 mm dishes until 90–95% confluency. For AT13387 treatment, cells were incubated with either 2 µM AT13387 or vehicle (saline) and after 4 h were exposed to HCl (0.02 N) for 1h before protein isolation. To stop the experiment, dishes were placed on ice and washed 3 times with ice-cold PBS. PBS was removed, and ice-cold lysis buffer was added (RIPA with protease inhibitor cocktail 1:100). Cells were scraped and the cell suspension was transferred to a microcentrifuge tube. Tubes were placed at 4 °C for 30 min under continuous agitation. Protein concentration was estimated by the BCA protein assay. Equal volumes of tricine buffer with 2% 2-mercaptoethanol were added to samples containing equal amounts of protein. Proteins were denatured by 10 min boiling at 100 °C. Protein lysates were then analyzed by Western blotting.

### 2.11. Endothelial Barrier Function

HLMVEC were seeded on electrode arrays (8W10E+), and endothelial barrier integrity was estimated by the electric cell-substrate impedance sensing (ECIS) technique, using an ECIS-Z theta instrument (Applied BioPhysics, New York, NY, USA). Experiments were conducted when a stable resistance was reached and maintained above 800 W, as we have previously published [21]. Experiments were performed in triplicates and repeated at least three times. Resistance values were collected and normalized to each well’s value at t = 0. Data are presented as means ± SEM.

### 2.12. Statistical Analysis

The results are presented as means ± standard error of the mean. Statistical significance of differences among groups was determined by one- or two-way analysis of variance (ANOVA), followed by the Tukey’s post-hoc test. Statistical analysis utilized GraphPad Prism Software (GraphPad Software, San Diego, CA, USA). The significance level was set at 0.05.

## 3. Results

### 3.1. AT13387 Modulates HCl-Induced Persistent Alveolar Inflammation

**Figure 1:**
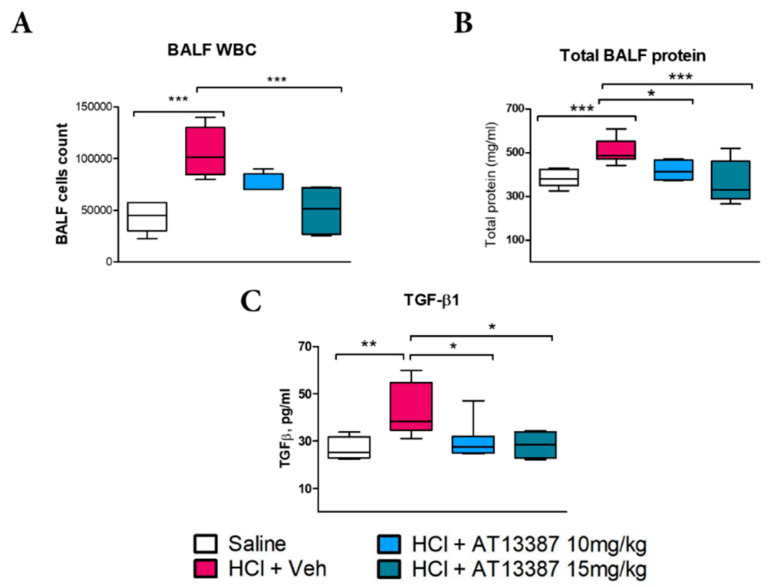
AT13387 blocks persistent alveolar cellularity, proteinosis and inflammation. (**A**) WBC, (**B**) Total protein and (**C**) TGF-β concentrations in bronchoalveolar lavage fluid (BALF) at 30 days post HCl instillation and treatment with AT13387 at 10 and 15 mg/kg 3×/week. *n* = 5 mice per group; *: *p* < 0.05; **: *p* < 0.01,***: *p* < 0.001, with 1-way ANOVA and Tukey’s post-test.

HCl produced persistent alveolar inflammation characterized by increased cellularity, proteinosis, and inflammatory cytokines, consistent with previously published results [10]. Treatment with 15 mg/kg AT13387, but not 10 mg/kg, significantly reduced WBC infiltration to control levels (Figure 1A). However, both 10 and 15 mg/kg AT13387 were able to reduce alveolar proteinosis (Figure 1B) and transforming growth factor-β (TGF-β) levels in BALF (Figure 1C).

### 3.2. AT13387 Ameliorates Pulmonary Fibrosis and NLRP3 Staining after HCl Exposure

**Figure 2:**
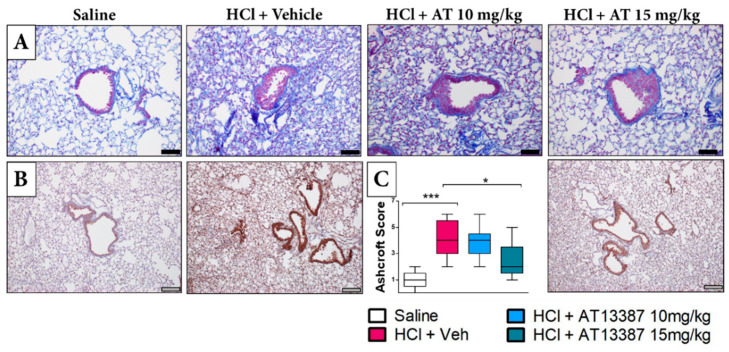
Subcutaneous treatment with AT13387 ameliorates pulmonary fibrosis. (**A**) Masson’s Trichrome staining of lung sections from mice instilled with vehicle (saline) or HCl and treated with AT13387 at 10 and 15 mg/kg or vehicle. (**B**) Immunohistochemical staining for the inflammasome NLRP3. (**C**) Quantification of chronic lung injury and pulmonary fibrosis performed by the Ashcroft score. Original magnification 10×–20×; black scale bars correspond to 50 µm, and grey scale bars correspond to 100 µm. *n* = 5 mice per group; *: *p* < 0.05; ***: *p* < 0.001, with 1-way ANOVA and Tukey’s post-test.

HCl exposure incurred profound changes in lung architecture, including increased thickness of alveolar structures, and infiltration of leukocytes. Masson’s trichrome staining identified increased deposition of collagen (Figure 2A). Similarly, HCl caused increased immunostaining for the inflammasome NLRP3 compared to saline-instilled animals (Figure 2B). Treatment with 15 mg/kg of AT13387, but not 10 mg/kg, reduced the histological evidence of fibrosis, as quantified by the Ashcroft score (Figure 2C).

### 3.3. AT13387 Blocks Pro-Fibrotic Pathways

**Figure 3:**
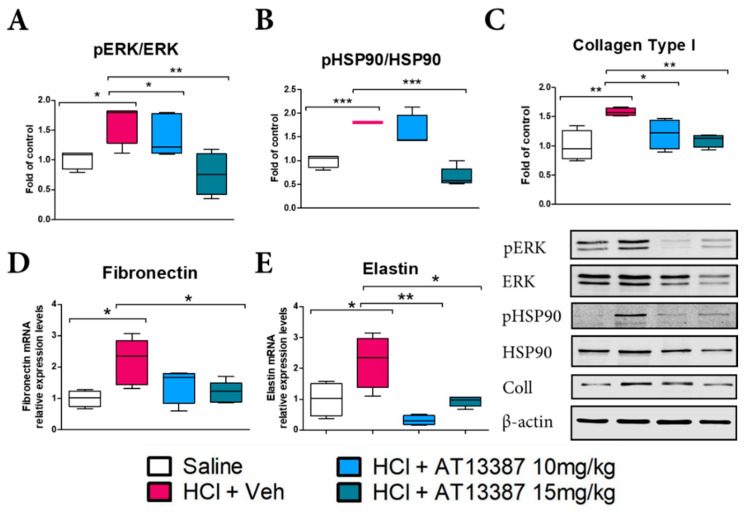
AT13387 prevents HCl-mediated fibrotic signaling and extracellular matrix deposition. AT13387 (at 10 or 15 mg/kg) reduced the activation (phosphorylation) of ERK1/2 (**A**), HSP90; (**B**), (15 mg/kg only), and Collagen (**C**) analyzed by Western blotting. All bands were normalized to β-actin. Relative expression levels of Fibronectin (**D**) and Elastin (**E**) mRNA were analyzed by RT-PCR. *n* = 4–5 mice per group; *: *p* < 0.05; **: *p* < 0.01, ***: *p* < 0.001, with 1-way ANOVA and Tukey’s.

We then measured the activity of critical pathways involved in profibrotic signaling and quantified the expression of extracellular matrix proteins. HCl evoked activation (phosphorylation) of extracellular signal-regulated kinase (MAPK/ERK) that was blocked by treatment with subcutaneous AT13387 at 10 and 15 mg/kg (Figure 3A). AT13387, at 15 mg/kg, also completely blocked the HCl-induced activation of HSP90 (Figure 3B). Blockage of these signaling pathways resulted in decreased deposition of Collagen at both 10 and 15 mg/kg AT13387 (Figure 3C). AT13387 also inhibited the HCl-induced overexpression of fibronectin and elastin mRNA at 30 days post-HCl instillation (Figure 3D,E).

### 3.4. AT13387 Prevents HCl-Induced Lung Dysfunction and Airway Hyper-Responsiveness to Methacholine

**Figure 4:**
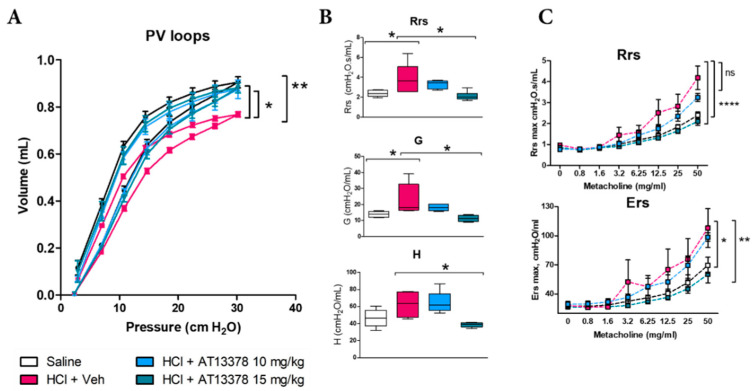
HCl causes lung dysfunction and airway hyperreactivity, blocked by AT13387. (**A**) Pressure–Volume relationships in mice after HCl instillation and treatment with vehicle or AT13387 s.c. 3×/week at 10 or 15 mg/kg. (**B**) Total Respiratory System Resistance (Rrs), tissue damping (G), and tissue elastance (H). (**C**) Rrs and total Respiratory System Elastance (Ers) were also evaluated in response to increasing concentrations of aerosolized methacholine. All studies were performed at 30 days after HCl instillation. *n* = 4–5 mice per group; ns: not significant, *: *p* < 0.05; **: *p* < 0.01, ****: *p* < 0.0001 with 2-way ANOVA and Bonferroni’s post-test (**A**), (**C**) or 1-way ANOVA and Tukey’s (**B**).

Mice instilled with HCl displayed severe abnormalities in lung mechanics as measured by Flexivent. HCl produced a downward shift in pressure–volume relationships (PV loops) indicative of airway dysfunction (Figure 4A) and increases in total respiratory system resistance (Rrs), tissue damping (G), and tissue elastance (H), all signs of increased lung stiffness (Figure 4B). Treatment with AT13387, at either 10 or 15 mg/kg, prevented the downward shift in PV loops caused by HCl, while only treatment with the higher dose was able to prevent increases in Rrs, G and H (Figure 4A,B). HCl is a well-known irritant and caused airway hyperresponsiveness to methacholine, which was prevented only by treatment with AT13387 at 15 mg/kg (Figure 4C).

### 3.5. AT13387 Prevents HCl-Mediated Endothelial Barrier Dysfunction

**Figure 5:**
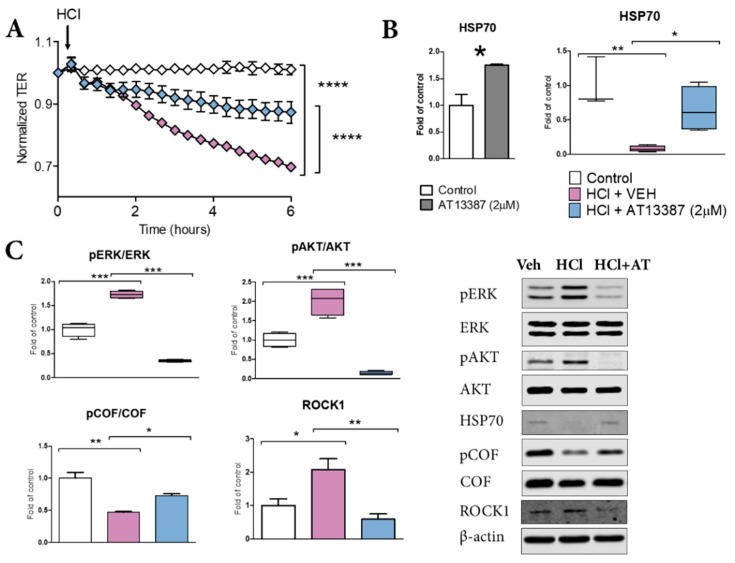
AT13387 blocks HCl-induced loss of endothelial barrier integrity. (**A**) Human Lung Microvascular Endothelial Cells (HLMVEC) were grown on gold electrodes (8WE10+) till stable resistance (>800 Ω) was achieved, pre-treated with vehicle or 2 µM AT13387 for 4 h, then challenged with 0.02N HCl. (**B**) HSP70 levels in control cells and in cell incubated for 4 h with 2 µM AT13387; HSP70 levels in cell pretreated with AT13387 2 µM or vehicle and challenged with HCl 0.2 N. (**C**) pERK1/2, pAKT/AKT, p-Cofilin and ROCK1 in HLMVEC pre-treated with 2 µM AT13387 for 4 h and then exposed to HCl for 1 h were analyzed by Western blotting. Data is represented in box and whickers plots or in bar graphs with means ± SEM; *n* = 3–4 per group; *: *p* < 0.05; **: *p* < 0.01, ***: *p* < 0.001, ****: *p* < 0.0001 with 2-way ANOVA and Bonferroni’s (**A**), 1-way ANOVA and Tukey’s (**B**,**C**).

Treatment with AT13387 was able to prevent HCl-mediated alveolar proteinosis at 30 days post-instillation, in vivo (Figure 1B). Thus, we investigated if this effect was related only to the anti-inflammatory/anti-fibrotic profile of AT13387, or, similarly to other HSP90 inhibitors, to a direct effect on endothelial barrier function [21]. Indeed, AT13387 (2 µM) prevented the loss of endothelial barrier integrity provoked by 0.02 N HCl (Figure 5A). AT13387 further prevented HCl-induced phosphorylation of ERK and AKT, with consequent mobilization of cytoskeletal proteins ROCK1 (Figure 5C). These effects were associated with AT13387-mediated increased levels of HSP70 (Figure 5B) and prevention of HCl-induced HSP70 decrease and cofilin dephosphorylation (Figure 5B,D).

## 4. Discussion

In this study, we investigated the use of a second-generation HSP90 inhibitor, AT13387, as a potential antidote against chronic lung injury and pulmonary fibrosis induced by exposure to HCl. HSP90 inhibitors are FDA-approved drugs under clinical investigation for cancer, but many are being repurposed as novel therapeutics for lung, renal, hepatic, and skin fibrosis [22,23,24]. In cancer studies, AT13387 has been employed at doses of 40, 60, and 70 mg/kg [25,26]. Here we discovered that treatment with a much lower dose of AT13387 (10 or 15 mg/kg) was well tolerated over a prolonged experimental period and effective in blocking the development of chronic lung injury. HSP90 inhibitors exert their beneficial effects via multiple mechanisms.

HSP90 inhibitors modulate inflammation, by preventing NF-κB translocation [27] and also by blocking NLRP3 activation [28]. In addition, they modulate TGF-β signaling, thus reducing fibrosis [13,29]; HSP90 inhibitors degrade TGF-β receptors, block nuclear translocation of Smad proteins, and inhibit Raf signaling, the non-canonical pathway of TGF-β [22,30,31]. Other HSP90 inhibitors, AUY-922 and 17-AAG, were previously shown to block TGF-β signaling in either a model of nitrogen-mustard induced chronic lung injury or in in vitro studies on cultured fibroblast [12,29]. In this study, AT13387 reduced TGF-β levels and those of pERK1/2 (Figure 1 and Figure 3).

HSP90 inhibitors also possess the ability to disrupt vesicular secretory trafficking of the extracellular matrix (ECM), thus reducing fibronectin deposition and tissue remodeling [32]. As shown by in vitro co-immunoprecipitation assay, HSP90 directly binds fibronectin and pharmacological or genetic HSP90 inhibition blocks fibronectin release from cells [33]. In agreement, treatment with AT13387 resulted in reduced deposition of collagen, fibronectin, and elastin and greatly attenuated the histological evidence of fibrosis (Figure 2). The inflammasome NLRP3 critically participates in IL-1β production during innate immune response and lung injury. Treatment with AT13387 reduced NLRP3 expression in the lungs (Figure 2).

HSP90 is a key regulator of alveolo-endothelial barrier function. The HSP90 inhibitor 17-AAG prevented LPS-induced endothelial hyperpermeability by blocking RhoA signaling and consequent myosin light chain 2 activation (MLC2) [34]. We previously showed that AUY-922 restored HCl-induced increased permeability by preventing RhoA activation and cytoskeletal rearrangements [21]. Here we demonstrate that pre-treatment with AT13387 protects HLMVEC from an even higher dose of HCl (0.02N) by promoting HSP70 expression and restoring cofilin function (Figure 5).

HSP70 acts as a co-chaperone of HSP90 and through the mediation of Hop/Stip regulates proteostatic balance, redirecting damaged proteins to degradation [35]. HSP70 also exhibits important cytoprotective properties, as it reduces hypoxia/reoxygenation injury [36], promotes cytoskeletal reassembly after gliadin exposure in a model of celiac disease [37], and prevents humidity- and heat stress–mediated apoptosis in cardiomyocytes [38]. Intracellular HSP70 lies in the cytoplasm, where it forms a complex with heat stress factor 1 (HSF-1) [39]. During HSP90 inhibition, HSF-1 is released, aggregated, and translocated to the nucleus, where it binds DNA and promotes the expression of HSPs through heat shock element (HSE) [40], thus explaining the collateral beneficial effects of HSP70 overexpression [41].

The study has several limitations. Although we analyzed proteins and RNA expression levels in lung tissue homogenates, cell population-based analysis could help better clarify how HSP90 inhibitors exert their beneficial effect. Also, as AT13387 modulates several pathways, proteomic studies could detect in vivo other target proteins that are involved in the therapy of pulmonary fibrosis. Further, we identified how restoration of HSP70 in vitro, by AT13387, is associated with restoration of endothelial barrier function, but further studies, in different animals’ models of lung injury (e.g., Bleomycin, LPS), are required to understand the combined efficacy of HSP90 inhibitors, during overexpression or inhibition of HSP70.

Most of the studies performed with HSP90 inhibitors use compounds that require parenteral administration and short-acting half-life. Here, we presented evidence that treatment with HSP90 inhibitor AT13387 (Onalespib) administered subcutaneously 3 times per week dramatically prevented the long-term effects of HCl exposure, suggesting its use in pre-hospital settings and disadvantageous situations.

## 5. Conclusions

HSP90 inhibitors represent a valid therapeutic approach for chronic lung injury and employed at lower doses are beneficial for cancer. AT13387 can be administered subcutaneously and due to its long half-life represents an innovative countermeasure.

## Data Availability

Derived data supporting the findings of this study are available from the corresponding author on request.
